# Longitudinal ^18^F-FDG PET and MRI Reveal Evolving Imaging Pathology That Corresponds to Disease Progression in a Patient With ALS-FTD

**DOI:** 10.3389/fneur.2019.00234

**Published:** 2019-03-19

**Authors:** Venkateswaran Rajagopalan, Erik P. Pioro

**Affiliations:** ^1^Department of Electrical and Electronics Engineering, Birla Institute of Technology and Science Pilani, Hyderabad, India; ^2^Department of Biomedical Engineering, Lerner Research Institute, Cleveland Clinic, Cleveland, OH, United States; ^3^Department of Neurology, Neurological Institute, Cleveland Clinic, Cleveland, OH, United States; ^4^Department of Neurosciences, Lerner Research Institute, Cleveland Clinic, Cleveland, OH, United States

**Keywords:** ALS-FTD, MRI, PET, cortical thickness, cortical area, aphasia

## Abstract

Single time point positron emission tomography (PET) studies of patients with amyotrophic lateral sclerosis and frontotemporal dementia (ALS-FTD), have demonstrated hypometabolism or hypermetabolism in certain brain regions. To determine whether longitudinal (at baseline and 20.4 months later) PET and magnetic resonance imaging (MRI) reveal evolving brain imaging pathology corresponding to clinical progression in a patient with ALS-FTD, cerebral glucose metabolic rate, cortical thickness (CT) and cortical area (CA) were obtained and symmetric percent change (SPC) for each calculated. The patient had worsening symptoms and signs of bulbar-onset upper motor neuron-predominant ALS as well as language and behavioral dysfunction. At baseline, minimally decreased ALSFRS-R (42/48) reflecting bulbar dysfunction was observed, along with language and executive function difficulties. At follow-up, bulbar and limb function rapidly declined as revealed by lower ALSFRS-R (27/48) and worsening language and cognitive function. PET revealed either hyper- and hypo-metabolic changes in several brain regions, especially in the left hemisphere. Marked clinical decline was accompanied by worsening cerebral and subcortical hyper and hypo-metabolism along with CT changes in regions known to degenerate in the primary progressive aphasia (PPA) form of ALS-FTD. Our case report demonstrates the progressive functional and structural neuroimaging abnormalities underlying clinical motor and neurocognitive deficits evolving in a patient with bulbar-onset ALS-FTD. Correlating neurological and neurocognitive decline with PET and MRI neuroimaging measures can provide better insights into pathophysiological mechanisms of ALS and ALS-FTD.

## Background

Growing evidence suggests that 18-fluorine-2-fluoro-2-deoxy-D-glucose (^18^F-FDG) positron emission tomography (PET) imaging of brain could play an important role in diagnosing and monitoring amyotrophic lateral sclerosis (ALS) (1). Studies using brain ^18^F-FDG PET have shown promise in differentiating ALS not only from controls but also from neurological conditions mimicking ALS (2). A large cohort study of ALS patients demonstrated that brain ^18^F-FDG PET imaging could differentiate even between ALS subtypes, including spinal-onset and bulbar-onset ALS (1). In addition to its use in the diagnosis of ALS, PET imaging could potentially shed light on underlying pathophysiological changes. For example, several studies in ALS (3–7) have suggested that brain hypometabolism represents neuronal degeneration, whereas hypermetabolism represents neuroinflammation and gliosis (i.e., astrocytosis and/or microgliosis). Turner et al. (8) observed decreased [^11^C] flumazenil binding in anterior and orbitofrontal regions in ALS patients with D90A SOD1 mutation. Others observed that brains of ALS patients with frontotemporal dementia (FTD) were hypometabolic in frontal and temporal regions (9, 10) or prefrontal and posterior cingulated cortices (11). These kinds of studies suggest that PET imaging can non-invasively evaluate cellular metabolism in brains of patients with ALS and ALS-FTD and potentially identify useful biomarkers of disease.

Cross sectional ^18^F-FDG PET studies in ALS patients with or without FTD, have demonstrated hypometabolic or hypermetabolic brain regions (9). In addition, cerebral glucose metabolic rate (CMR_glc_) abnormalities have been detected in both motor and extra-motor regions of non-demented ALS patients with spinal- and bulbar-onset disease (1), with the patterns of abnormality differentiating the two onset subtypes. Our recent study revealed hypometabolism of relevant cortical areas in ALS-FTD patients compared to controls (12).

All the above studies were cross-sectional in nature including that of Pagani et al. (1), which examined a large (*n* = 95) cohort of ALS patients. In order to better define the sensitivity and specificity of PET imaging in the diagnosis of ALS these authors recommended longitudinal studies. Most of the above PET imaging studies in ALS did not correlate disease changes of metabolic function with brain structure (1, 6, 13). ^18^F-FDG PET studies alone cannot determine whether metabolic changes precede or follow structural ones. Although cross-sectional, our previous study of ALS-FTD patient brains revealed that regions of cortical hypometabolism overlapped structural abnormalities and appeared to precede them in some regions (12). Reflecting the non-specific nature of nuclear metabolic imaging, different underlying pathophysiological changes in ALS can result in similar ^18^F-FDG PET changes, for e.g., hypermetabolism can result from neuroinflammation, astrocytosis, or both.

To address the above limitations, this study aimed to examine how brain metabolism and structure change longitudinally in a patient with bulbar-onset ALS-FTD. We wished to evaluate hypo- and hypermetabolism changes in ALS in the context of structural changes (cortical thickness [CT], cortical surface area [CA]), which can reflect evolving neurogliosis and neurodegeneration.

## Case Presentation

### Neurological and Motor Deficits

A 69 year-old right-handed Caucasian female was evaluated for a 15 month history of cognitive and language impairment, slurred speech, and mild weakness. Her first symptom was difficulty “getting the words out” even though she knew what she wanted to say or write. Very shortly thereafter, her voice became “strangled” sounding and progressively slurred. Interestingly, the patient denied any problems with her speech or ability to express herself until only about 2 months prior to the initial evaluation.

Past history was significant for hypertension, mixed hyperlipidemia, osteoporosis, gastroesophageal reflux, cholecystectomy, and carpal tunnel syndrome release. There was no history of psychiatric disturbances, sleep problems, and drug or alcohol abuse. Although herself a non-smoker, she had been exposed to second hand smoke during her 40-year marriage to a heavy smoker. She was exposed to a variety of chemicals and toxins working as a beautician for 40 years. There was no family history of neuromuscular disorders, including motor neuron disease or ALS, Parkinson's disease, multiple sclerosis, or dementia. Medications included and antihypertensive, antacid, and anxiolytic.

Laboratory investigations revealed normal CBC and differential, comprehensive metabolic profile, TSH, CRP, but elevated cholesterol and triglycerides. Serum proteins were normal except for slightly reduced IgG consistent with mild hypogammaglobulinemia of no clinical relevance. Mild secondary hyperparathyroidism was identified and paraneoplastic antibodies (anti-Hu, anti-Yo/Purkinje cell) were negative. Testing for *C9orf72* gene expansion (on previously banked DNA) was negative. Electrodiagnostic testing of the right body, including cranial muscles, revealed no evidence of lower motor neuron degeneration.

At baseline evaluation, neurologic deficits included lack of insight, pseudobulbar affect, severe dysphasia, dysarthria with right body predominant upper motor neuron (UMN) signs and minimally reduced revised ALS functional rating scale (ALSFRS-R) score only in the bulbar domain (6/12/12/12 = 42/48). Formal psychometric testing (see below) revealed significant expressive language problems, with mild complex attention and executive function difficulties.

At follow-up evaluation 20.4 months later, neurologic decline was primarily bulbar (mixed UMN and lower motor neuron [LMN] signs), but also involved limb and respiratory functions, as reflected by decreased ALSFRS-R score across all domains (1/10/8/8 = 27/48). Rate of decline of the ALSFRS-R score over that period of time was substantial [ΔFS = −0.74, derived when the difference between the two ALSFRS-R total scores (14) is divided by 20.4 months].

### Neurocognitive Deficits

Formal neuropsychometric testing was performed ~20 months apart (at about the same time as neuroimaging) using the same standardized tests at both time points, with some modifications at follow-up testing because of significant progression in deficits. These tests included: Wechsler Adult Intelligence Scale, 3rd Ed. (Information, Block Design, Digit Symbol, Matrix Reasoning and Symbol Search), Peabody Picture Vocabulary Test (Form IIIA), Mattis Dementia Rating Scale (Construction, Conceptualization), Boston Naming Test, Token Test, Aural Comprehension, Victoria Symptom Validity Test, Wechsler Memory Scale, 3rd Ed. (Faces), Spatial Span, Rey Auditory Verbal Learning Test, Trail Making Test (Forms A & B), Visual Form Discrimination, Figural Fluency, and Wisconsin Card Sorting Test.

At baseline, the patient's performance was most notable for expressive language dysfunction. She performed poorly on a measure of confrontation naming, and qualitatively, her written statements were notable for word omissions and substitutions. The patient also had difficulty on a measure of verbal complex attention and mild difficulties on measures of set-shifting. Her performance on other neuropsychological measures, including those assessing receptive language abilities, generally fell within expectation. She did not endorse significant emotional distress but was rather defensive when responding to self-report measures, which might have suppressed these results.

Compared with the baseline neuropsychological evaluation, at follow-up (20.4 months later), the patient showed a marked decline in her language and executive functions. Both expressive and receptive language capabilities declined, meaning that the patient did not reliably comprehend the syntax in sentences and was showing signs of difficulty identifying concrete nouns and verbs. Her written expression declined to a non-functional level at this point, due largely to her dysgraphia; while she appeared to know what she wanted to write, she was unable to reliably write out the word she was searching for and the result was unintelligible. The patient's memory performance and visuospatial skills showed remarkable stability when compared with the previous evaluation, as long as language function was not involved. Emotionally, the patient denied depressive symptoms on a questionnaire (though her comprehension of this was not clear) and she did not show overt signs of depression or anxiety. Over the 20.4 month period, marked decline occurred in executive functions, language (both expressive and receptive), resulting in aphasia and dysgraphia. The pattern of cognitive decline was consistent with worsening FTD, which had produced aphasia (in addition to the patient's anarthria), and was most consistent with a primary progressive aphasia (PPA) form of ALS-FTD.

## Investigations and Tests

High-resolution T1-weighted 1.5T magnetic resonance imaging (MRI) and ^18^F-FDG PET images were acquired during routine clinical evaluation as previously described (12) on the same day at baseline (15 months after symptom onset) when the patient was 69 years-old, and 20.4 months later when she was 71 years old. The data for this study were part of our routine clinical neuroimaging evaluation, which was approved by the Institutional Review Board at the Cleveland Clinic to be stored and analyzed as de-identified images (adhering to HIPAA regulations) after the patient provided verbal consent in accordance with the Declaration of Helsinki (1991). Written informed consent was obtained for publication of this Case Report from the patient's legal representative. At each time point, measures of CT, CA and CMR_glc_ were obtained and symmetrized percent change (SPC) for each calculated.

### Gray Matter Cortical Thickness Analysis

Longitudinal CT alterations were estimated using openware Freesurfer 5.1. (http://surfer.nmr.mgh.harvard.edu/). Images at each time point were processed in the following manner: images were first motion corrected and skull stripped (15), segmented followed by automated topology estimation and correction, and GM-WM and GM-CSF boundaries optimally placed. Deformable procedures were then performed and cortical models such as surface inflation and registration to a spherical atlas (14) were completed. We utilized longitudinal routines that included creation of a base template to which the images from two time points were then registered, as detailed elsewhere (16). SPC maps of CT and CA were finally obtained, which map differences in these values between the two time points divided by the average CT and CA. An example of SPC map calculation for CT is given below (16).

(1) $$\begin{array}{l} Average\;thickness=\frac{\left(thickness\;at\;time\;pt\;1+\;thickness\;at\;time\;pt\;2\right)}{\text{2}} \end{array}$$

(2) $$\begin{array}{l} Rate\;of\;change=\frac{\left(thickness\;at\;time\;pt\;2-\;thickness\;at\;time\;pt\;1\right)}{\text{time pt }2-\text{ time pt }1} \end{array}$$

(3) $$\begin{array}{l} Symmetrized\;percent\;change\;\left(SPC\right)=\frac{100\times rate\;of\;change}{average\;thickness} \end{array}$$

The SPC surface maps were then converted into volume files, which were brought to MNI space to identify significant regions based on the AAL atlas.

### PET Imaging Analysis

^18^F-FDG PET images were processed using openware SPM using the following steps: (1) estimate and normalize, (2) smoothing, and (3) calculation of SPC map. Specifically, PET images at each time point were registered linearly and non-linearly to the Dementia-Specific [18F]-FDG-PET template from 100 subjects in MNI space (17) using estimate and normalize option. Subsequently, the images were smoothed using a Gaussian kernel of 8 mm and finally, SPC map of change in metabolism was calculated similar to SPC map calculation for CT. The resulting positive or negative SPC values represented brain regions that were either hypermetabolic or hypometabolic, respectively. All brain regions showing metabolic changes were identified with the AAL MNI atlas.

## PET and MRI Findings

**Figure 1:**
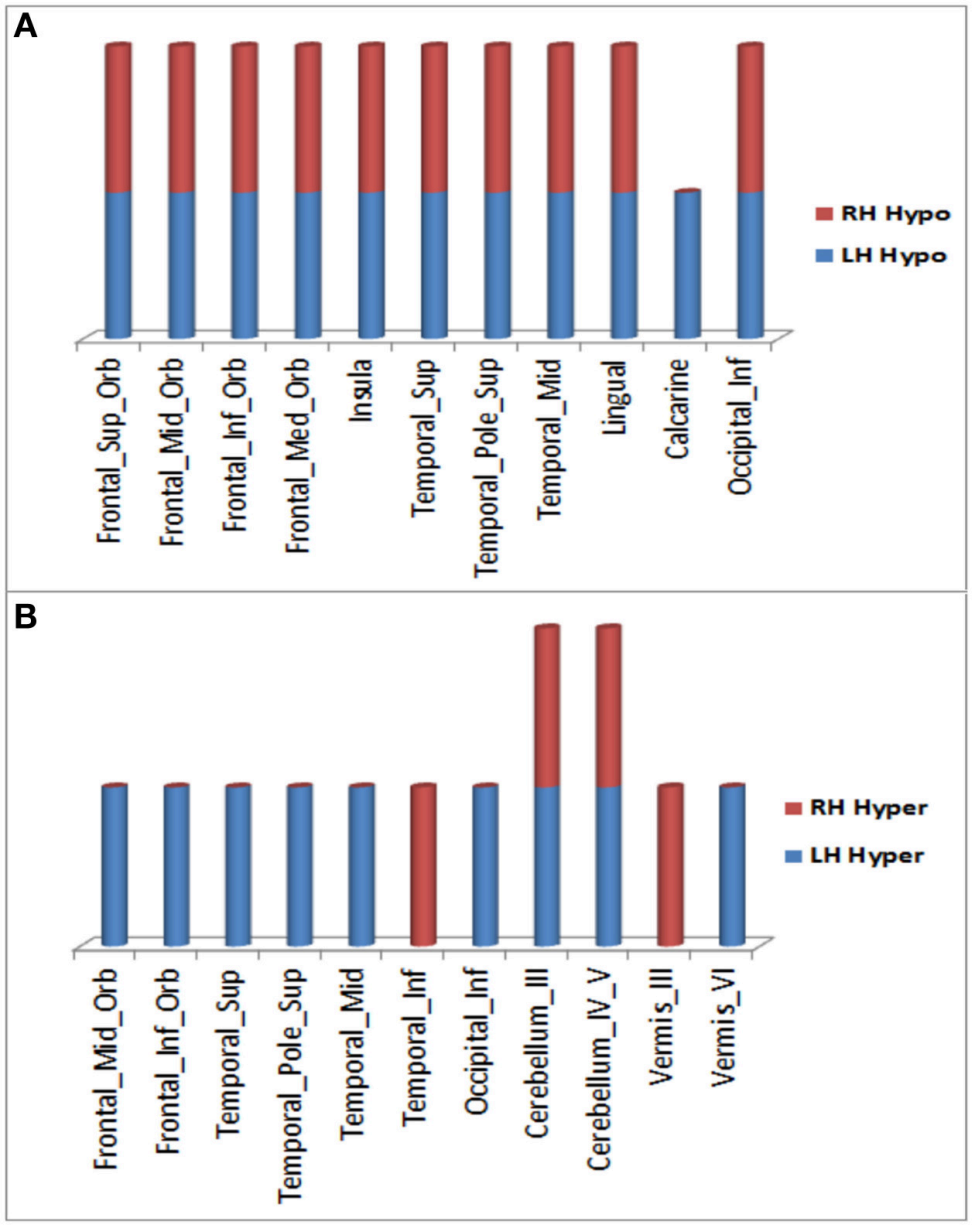
Brain regions in right and left hemispheres of the ALS-FTD patient where significant worsening of ^18^F-FDG PET hypometabolism **(A)** or hypermetabolism **(B)** occurred over 20.4 months.

Over the 20.4 month period of clinical decline, worsening of hypometabolism was observed in multiple brain regions (Figure 1A) of (1) both right and left hemispheres: superior frontal gyrus (orbital part), middle frontal gyrus (orbital part), inferior frontal gyrus (orbital part), medial frontal gyrus (orbital part), insula, superior temporal gyrus (including temporal pole), middle temporal gyrus, lingual gyrus, and inferior occipital gyrus; (2) left hemisphere only: calcarine cortex. In addition, worsening hypermetabolism was observed in multiple brain regions (Figure 1B) of (1) only left hemisphere: middle frontal gyrus (orbital part), inferior frontal gyrus (orbital part), superior temporal gyrus (including temporal pole), middle temporal gyrus, inferior occipital gyrus, and cerebellar vermis-VI; (2) both right and left hemispheres: cerebellum-III, cerebellum-IV,V, (3) right hemisphere only: inferior temporal gyrus, vermis-III. These metabolic changes involving forebrain and cerebellar regions were observed slightly more frequently in left than in right hemisphere regions (Figure 1). Demonstration of both hypo- and hypermetabolism in the same cortical region may reflect differences in connectivity with other brain regions or simply a limitation of ^18^F-FDG PET to resolve differential involvement of gyral subregions, among other explanations.

**Figure 2:**
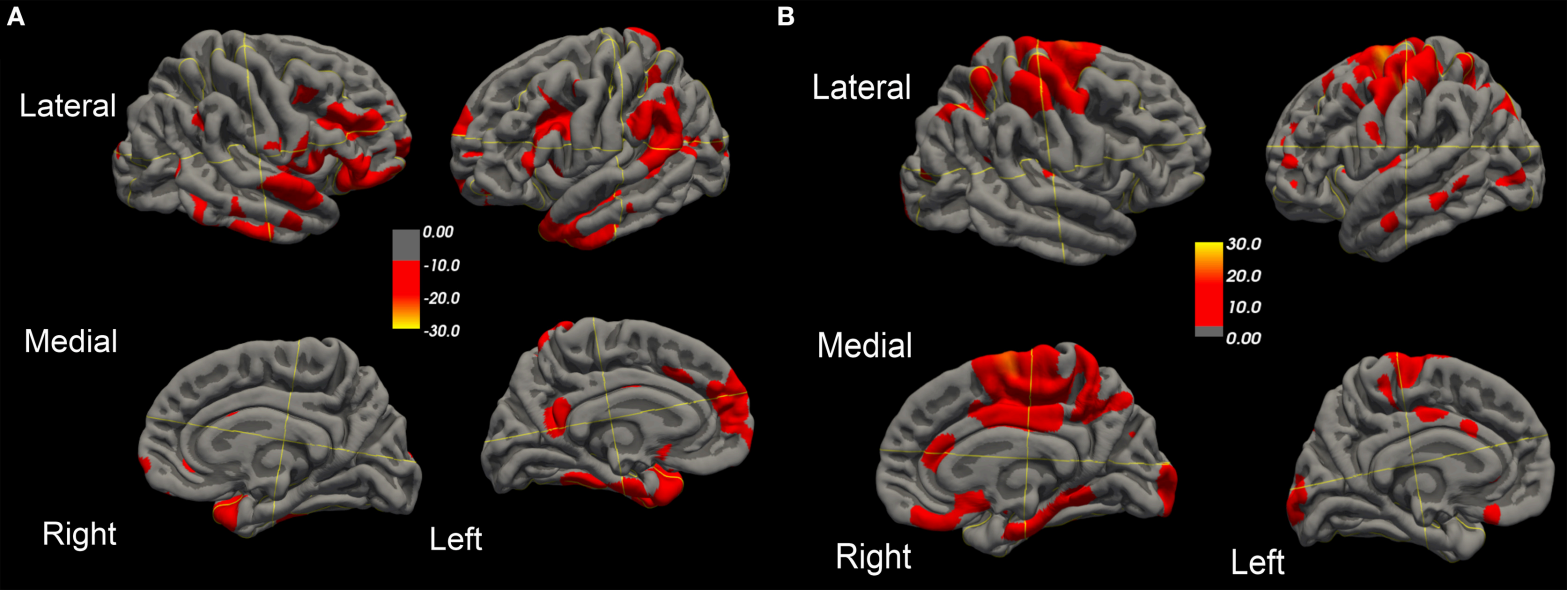
Surface-rendered MRI of right and left hemispheres show symmetrized percent change (SPC) values of cortical thickness decrease (cortical thinning) **(A)** or increase (cortical thickening) **(B)** in the ALS-FTD patient over 20.4 months.

**Figure 3:**
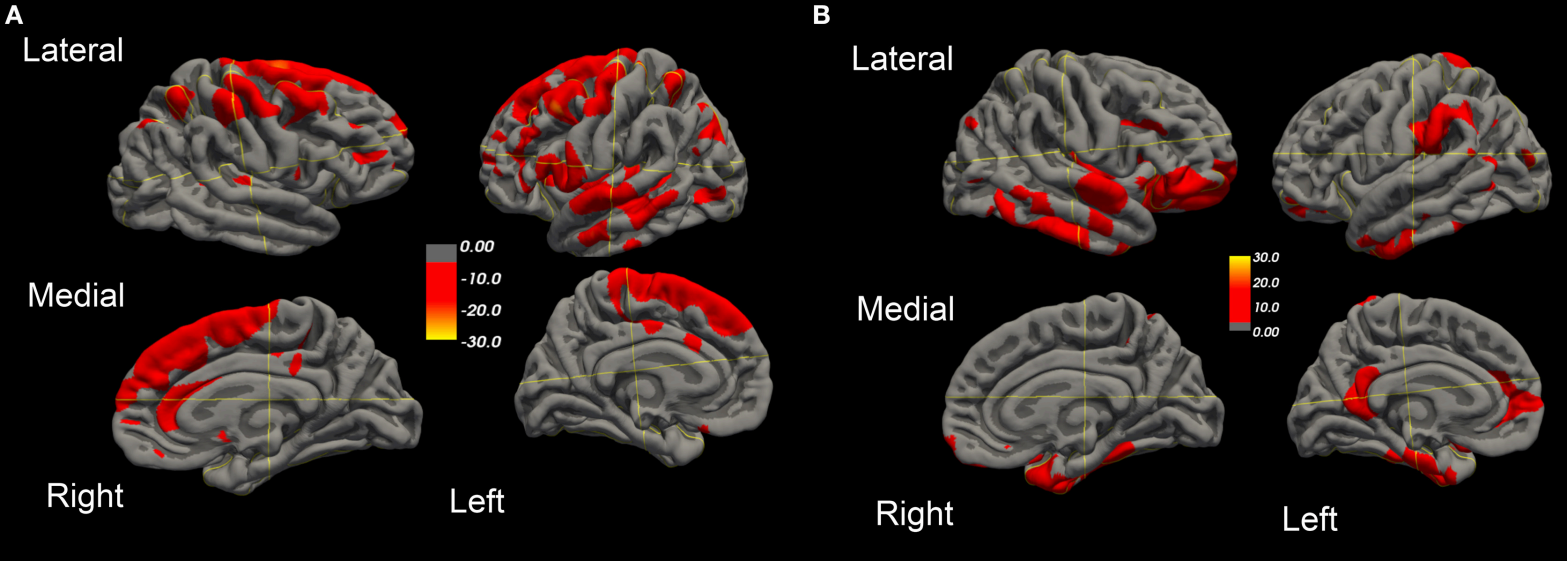
Surface-rendered MRI of right and left hemispheres SPC values of cortical surface area decrease **(A)** or increase **(B)** in the ALS-FTD patient over 20.4 months.

**Figure 4:**
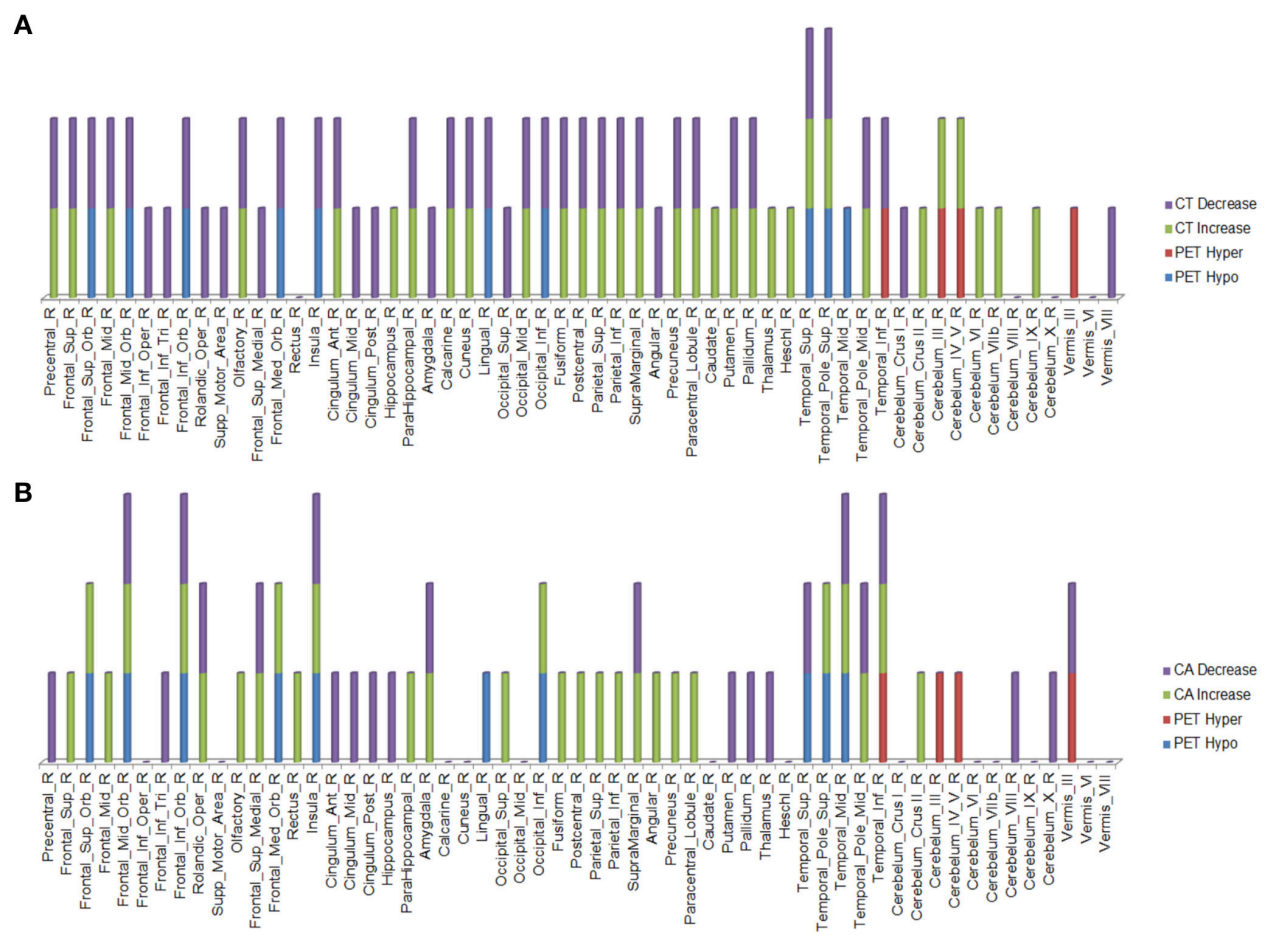
Right hemisphere brain regions with changes in cortical thickness (CT) **(A)** and cortical area (CA) **(B)** relative to alterations in ^18^F-FDG PET metabolism.

**Figure 5:**
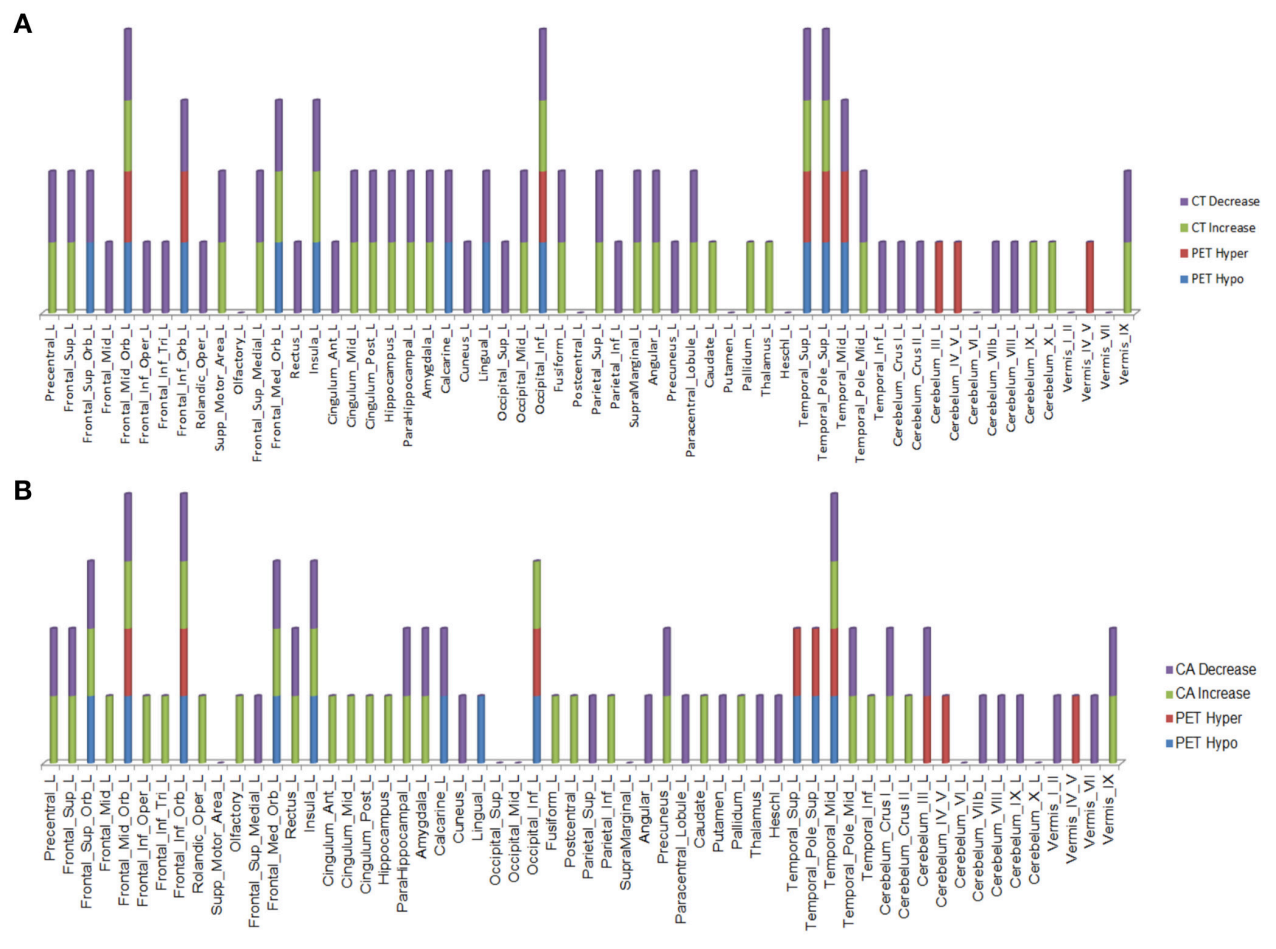
Left hemisphere brain regions with changes in cortical thickness (CT) **(A)** and cortical area (CA) **(B)** relative to alterations in ^18^F-FDG PET metabolism.

Over this same period of time, cortical thinning worsened in bilateral prefrontal lobe regions, including Broca's area, with significantly greater changes in left compared to right hemispheres in temporal pole, superior temporal, entorhinal, and parahippocampal cortices. Brain regions with changes in CT (thinning or thickening) and CA (decreased or increase) between baseline and 20.4 month time points studied are shown in Figures 2–5. Regional changes of CT (thinning or thickening), of CA (decrease or increase), and of CMR_glc_ (hypo- or hypermetabolism) are shown in right (Figure 4) and left (Figure 5) hemispheres.

## Discussion

Clinical decline over 20.4 months in this 69/71 year old patient was accompanied by worsening hypo- and hypermetabolism of cerebral and cerebellar regions, as well as cortical thinning in regions known to degenerate in the PPA form of ALS-FTD, consistent with her clinical diagnosis. Worsening hypometabolism (and hypermetabolism) was generally more prominent in the left compared to right hemisphere, which corresponded to the patient's worsening right body-predominant UMN clinical signs. This is consistent with findings in earlier studies of asymmetric hypometabolism in ALS-FTD patients (13, 18). Both motor and extra motor regions, which include prefrontal, frontal, temporal, subcortical, and cerebellar regions revealed hypo- or hyper- metabolism; several of these areas subserve cognitive, language, and behavioral functions, which demonstrated worsening by the 2nd time point. Worsening metabolism observed in frontal lobe in our study is in line with a previous FDG-PET-MRI study of an SOD1mutation-expressing familial ALS patient with normal cognitive testing except for impaired performance in the Rey-Osterrieth Complex Figure (ROCF) Test (19). The authors documented extension of hypometabolism over 6 months from the right supramarginal and middle frontal gyri to right frontopolar and caudate nucleus regions. Over that time period, atrophy of the right supramarginal gyrus detected on MRI remained, as did cognitive testing, including the ROCF Test result. Interestingly, worsening *hyper*metabolism was also observed in frontal, temporal, subcortical, and cerebellar regions of our patient with ALS-FTD. Brain hypermetabolism has been previously reported in ALS patients compared to controls at a single time-point (6), but our findings indicate a worsening change with disease progression. Although the mechanism of hypermetabolism in these regions is unclear, it could result from either compensatory activation (recruitment) of adjacent (motor or extramotor) regions or proliferation of astrocytes and microglia (gliosis) in response to cortical and subcortical degeneration.

In the regions of ^18^F-FDG PET hypo- or hypermetabolism, we also studied structural changes of CT and CA. As can be seen in Figures 4, 5, structural changes appeared to precede functional changes, including in the frontal lobe, precentral gyrus, middle temporal gyrus (including temporal pole), insula, cingulum, cuneus, lingual gyrus, occipital lobe, caudate, putamen, and thalamus (Figures 4, 5). On the other hand, functional changes preceded structural ones in the cerebellum, including vermis. Of note, all these affected brain regions play important roles in cognitive functioning. In addition, many brain regions revealed an *increase* (not decrease) in CA at the 2nd time point, which like the ^18^F-FDG PET findings, were more prominent in the left hemisphere. CA changes preceded ^18^F-FDG PET changes in the frontal lobe, precentral gyrus, postcentral gyrus, cingulum, angular gyrus, hippocampus, amygdala, putamen, and thalamus. Although the reasons for such divergent changes of CT and CA are unclear, these measures are likely influenced differentially by the numerous processes occurring during neurodegeneration. Previous studies of aging adult brains have shown that reductions in CT and CA are independent processes (20) and that they can be inversely related, i.e., as CT increases, CA decreases, and vice versa (21). In this study, we observed such an inverse relationship between these measures in several brain regions, including in the left hemisphere: middle frontal gyrus, inferior frontal gyrus (opercular part), inferior frontal gyrus (triangularis part), rolandic operculum, etc. A more recent longitudinal aging study (22) demonstrating an inverse relationship between CT and CA suggested that underlying neurobiological mechanisms differentially affect these structural measures. Although neuroinflammation and gliosis are known to follow neurodegeneration, they may be primary or even preceding events in certain cases or stages of ALS.

Based on our present findings and those of others (23), we hypothesize that neuroinflammation and gliosis may result in ^18^F-FDG PET hypermetabolism but differentially affect CT and CA. Qiu and colleagues propose that early stages of inflammation in major depressive disorder (e.g., release of proinflammatory cytokines) may activate astrocytes (comprising ~90% cortical tissue volume) to hypertrophy, proliferate, and extend and interdigitate their processes, which could thereby result in cortical thickening. The inverse relationship with CA, as discussed above, could result in reduction of cortical surface area.

We propose that the stage of the underlying neurodegenerative process determines the type and extent of structural (CT, CA) change in hypo- and hypermetabolic brain regions. Therefore, ^18^F-FDG PET metabolic imaging without structural imaging does not provide as much information about ALS-related neurodegenerative changes as do both together. Interpretations from this longitudinal study of a single subject study are limited and considered preliminary; more ALS patients studied in a similar manner are recommended to confirm our findings.

## Conclusion

Longitudinal ^18^F-FDG PET revealed both hypo- and hyper metabolic changes in several brain regions of an ALS-FTD patient with disease progression, which were accompanied by MRI-revealed structural changes. Determining relative proportions of metabolic vs. structural changes in each affected brain region may reveal their sequential progression. While worsening hypometabolic changes likely represent neuronal degeneration, hypermetabolic changes probably arise from inflammation and/or astrogliosis, which may be accompanied by differing structural changes of CT and CA. Although ^18^F-FDG PET imaging of cerebral glucose metabolism provides useful information of function, its combination with MRI-derived data mentioned above (as well as other structural measures) is complementary and adds more insight into the pathogenic mechanisms of ALS. Future longitudinal PET-MRI correlative studies of more patients may provide additional insights and confirm our findings.

## Data Availability

The datasets for this manuscript are not publicly available because data are property of the Cleveland Clinic Foundation. Requests to access the datasets should be directed to pioroe@ccf.org.

## Author Contributions

VR acquired data, processed data, analyzed the results, and wrote the manuscript. EPP designed the study, acquired data, analyzed the results, and made substantive revisions to the manuscript.

### Conflict of Interest Statement

EPP is holder of the Barry Winovich (Bright Side of the Road Foundation) Chair in ALS Research, and receives support from Samuel J. and Connie M. Frankino Charitable Foundation. He receives clinical trial and research funding from NIH/CDC, ALS Association, Iron Horse Diagnostics, Inc., and serves as consultant to Avanir Pharmaceuticals, Inc., Cytokinetics, Inc., ITF Pharma, Inc., MT Pharma America, Inc., and Otsuka America, Inc. The remaining author declares that the research was conducted in the absence of any commercial or financial relationships that could be construed as a potential conflict of interest.
